# The Spanish Version of the Western Ontario Meniscal Evaluation Tool Is a Reliable, Valid, Precise, and Highly Responsive Instrument for Patients With Meniscal Pathology at a Minimum 6‐Month Follow‐Up

**DOI:** 10.1002/ars2.70131

**Published:** 2026-10-23

**Authors:** Miguel Angel Palacios‐Flores, Maximiliano Ibañez, Zichun Liu Wang, Nicola Pizza, Raul Torres‐Claramunt, Simone Perelli, Joan Carles Monllau

**Affiliations:** ^1^ EsSalud ‐ Hospital Nacional Edgardo Rebagliati Martins Lima Peru; ^2^ Knee and Arthroscopy Unit, Institut Català de Traumatologia i Medicina de l’Esport (ICATME) Hospital Universitari Dexeus Barcelona Spain; ^3^ Hospital del Mar Barcelona Spain

## Abstract

**Purpose:**

To translate, culturally adapt, and validate the Western Ontario Meniscal Evaluation Tool (WOMET) into Peninsular Spanish and to evaluate its psychometric properties (reliability, validity, precision, and responsiveness) in patients undergoing knee arthroscopy for meniscal pathology.

**Methods:**

This retrospective study with prospectively collected data (from July to December 2025) followed international guidelines for cross‐cultural adaptation (forward‐backward translation, expert committee review, and pilot testing in 30 patients). The Spanish WOMET and Knee Injury and Osteoarthritis Outcome Score‐4 were assessed electronically at baseline (T0), retest (T1, 2 weeks later), and 3 (T2) and 6 (T3) months postoperatively (a minimum 6‐month follow‐up). Reliability was assessed with intraclass correlation coefficient; internal consistency with Cronbach's *α* and McDonald's *ω*; convergent validity with Spearman *ρ* against Knee Injury and Osteoarthritis Outcome Score‐4; precision with standard error of measurement and minimal detectable change at 95% confidence level; and responsiveness with standardized response mean.

**Results:**

Seventy‐one patients (67.7% men; median age 47 years, interquartile range 18‐60) with a mean follow‐up of 6.1 months (range: 5.7‐6.8) were evaluated. Test‐retest reliability was excellent (total WOMET intraclass correlation coefficient = 0.96, 95% confidence interval 0.94‐0.97). Internal consistency was high (*α* = 0.90, *ω* = 0.92). Convergent validity with Knee Injury and Osteoarthritis Outcome Score‐4 was strong (*ρ* = 0.90, *P* < .001). Precision was good (standard error of measurement = 2.78, minimal detectable change at 95% confidence level = 7.68). Responsiveness was substantial (standardized response mean = 2.71 at 3 months and 2.56 at 6 months; *P* < .001), exceeding values reported for most other language versions.

**Conclusions:**

The Spanish version of the WOMET questionnaire is a reliable, valid, precise, and highly responsive instrument for assessing clinical outcomes in patients with meniscal pathology treated surgically in Spanish‐speaking patients. Its brevity (16 items), specificity, and excellent patient acceptability make it an ideal tool for routine clinical practice and research.

**Level of Evidence:**

Level IV, retrospective case series.

## Graphical abstract

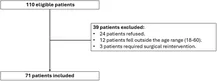

Meniscal pathology represents one of the most common conditions encountered in routine orthopaedic practice and is the leading cause of knee pain in the general population worldwide, with an incidence of 61 to 79 cases per 100,000 persons per year.^1^ In Spain, arthroscopic partial meniscectomy is estimated to be one of the most frequently performed knee procedures, with rates of 4.8 to 6.3 procedures per 10,000 inhabitants per year.^2^ Given the high prevalence of meniscal pathology and the need to assess treatment outcomes, various patient‐reported outcome measures have been developed. Among them, the Knee Injury and Osteoarthritis Outcome Score (KOOS) is a widely used knee‐specific instrument. However, it is not specifically designed for meniscal pathology. The Western Ontario Meniscal Evaluation Tool (WOMET) stands out as a disease‐specific questionnaire designed to evaluate outcomes in patients undergoing treatment for meniscal pathology.^3^ Unlike the KOOS, the WOMET assesses patients treated for meniscal pathology across 3 domains: physical symptoms, sports/recreation/work/lifestyle, and emotions. These domains provide a comprehensive evaluation and enable quantification of outcomes in patients with meniscal pathology, making them more sensitive and responsive in this specific population.

This score was originally validated in English^3^ and has subsequently been translated and validated into German,^4^ Dutch,^5^ Turkish,^6^ Danish,^7^ Chinese,^8^ Finnish,^9^ and, more recently, Italian.^10^ In all reported languages, it has shown excellent psychometric properties, undergoing not only linguistic translation but also cultural adaptation according to established guidelines,^11^, ^12^ yielding satisfactory results in terms of reliability, precision, validity, and responsiveness to change, along with high patient acceptability.

Within the context of the Spanish National Health System, the availability of validated and culturally adapted instruments is essential to objectively quantify clinical outcomes and treatment effectiveness in patients. Therefore, the purposes of this study were to translate, culturally adapt, and validate the WOMET into Peninsular Spanish and to evaluate its psychometric properties (reliability, validity, precision, and responsiveness) in patients undergoing knee arthroscopy for meniscal pathology. Our hypothesis was that the Spanish version of the WOMET score would exhibit psychometric properties comparable to those of previous versions, including high reliability, validity, precision, and responsiveness to clinically relevant changes in patients’ functional status.

## METHODS

This was a retrospective study with prospectively collected data, aimed at validating a clinical outcome questionnaire (WOMET). Data collection took place between July and December 2025 at 2 reference hospitals for knee surgery in Barcelona, Spain. This study was approved by the Ethics Committee of the participating institutions. All patients provided written informed consent before inclusion. The study was conducted in accordance with the ethical principles of the Declaration of Helsinki and its later amendments, as well as with the current Spanish legislation on biomedical research.

Patients were included if they were between 18 and 60 years of age, had sufficient cognitive and linguistic ability to understand and complete the questionnaires, and presented with isolated meniscal lesions (traumatic or degenerative) amenable to surgical repair or meniscectomy.^6^ Patients were excluded if they had concomitant ligamentous or chondral lesions (Outerbridge grade III‐IV),^5^ knee osteoarthritis (Kellgren‐Lawrence grade III or IV),^4^ previous surgery on the index knee (except simple diagnostic arthroscopy), or relevant comorbidities such as cancer, neuromuscular disorders, or inflammatory musculoskeletal diseases (e.g., rheumatoid arthritis).^6^

Surgery was indicated in patients with persistent mechanical symptoms (locking, catching, or giving way) and/or pain unresponsive to at least 3 months of conservative treatment (physical therapy, activity modification, and analgesics).

All procedures were performed arthroscopically by experienced knee surgeons using standard anterolateral and anteromedial portals. Partial meniscectomy was performed in cases of irreparable tears, whereas meniscal repair (all‐inside suture technique) was performed when the tear was located in the red‐red or red‐white zone and considered reparable.

### Translation and Cross‐Cultural Adaptation Process

The study followed the forward‐backward translation process for the WOMET score^3^ in accordance with internationally accepted guidelines.^11^, ^12^ The process included the following stages:^13^, ^14^

• Forward translation: 2 independent translators of Spanish origin produced separate versions. One was an orthopaedic surgeon experienced in knee pathology, and the other was a professional translator with no medical background to ensure clear and comprehensible wording for patients.
• Synthesis of translations: the 2 versions were discussed with the principal investigator to resolve discrepancies and produce a single reconciled translation.
• Backward translation: the synthesized version was blindly translated back into English by a native English speaker with no medical background to identify potential conceptual or semantic inconsistencies when compared with the original WOMET questionnaire.
• Expert committee review: the results were reviewed by a committee comprising the 3 translators involved in the process, reaching a consensus‐based final prepilot version.^4^, ^6^
• Pilot testing: the prefinal version was administered to 30 patients to identify any cognitive or culturally relevant issues, leading to the definitive Spanish version.

The sample size was determined by nonrandomized convenience sampling, based on recommendations for questionnaire validation studies suggesting 50 to 100 patients.^10^, ^15^ This provided sufficient statistical power for reliability, internal validity, and precision analyses while accounting for potential loss to follow‐up, as reported in previous studies.^4^, ^5^, ^6^, ^7^, ^8^, ^9^, ^10^

### Measurements

The Spanish WOMET questionnaire consists of 16 items across 3 domains (physical symptoms, sports/recreation/work/lifestyle, and emotions). These domains comprehensively reflect the functional impact of meniscal pathology on patients’ lives.^10^ Each item is scored using a visual analog scale from 0 to 100, where 0 represents no symptoms or impact and 100 represents the maximum degree of symptomatology or impairment in quality of life and emotions. The raw score is calculated by summing the 16 items (range: 0‐1600), subtracting this from 1600, dividing by 16, and inverting the result so that higher scores indicate better health (0 = worst; 100 = best).

The KOOS is a knee‐specific questionnaire assessing overall knee function,^16^ comprising 42 items distributed across 5 domains: symptoms, pain, activities of daily living, sport and recreation, and knee‐related quality of life.^10^, ^17^ The KOOS‐4 is a short version containing 25 items divided into 4 domains: symptoms, pain, sport and recreation, and quality of life.^10^, ^17^ Both versions use a Likert response scale ranging from “0” (no symptoms or no impact on the patient's life) to “4” (excessive symptoms or excessive impact on the patient's life); each item is transformed to a 0 to 100 scale. The maximum raw score for the KOOS‐4 is 2500 (range: 0‐2500); this is subtracted from the obtained score and divided by 25 to yield relative parameters from 0 to 100, which are subsequently inverted to maintain the conventional clinical direction (0 = worst; 100 = best). This questionnaire was used for comparison because it has been previously validated in Spanish^18^ and served as a comparator instrument to assess the construct validity of the Spanish version of the WOMET questionnaire.

Both questionnaires (WOMET and KOOS‐4) were administered electronically via Google Forms. They were completed at 4 time points:

• Preoperatively: in 2 stages—baseline measurement at diagnosis and surgical indication (T0) and retest measurement 2 weeks later (T1) to minimize recall bias.^19^
• Postoperatively: at 3 months (T2) and 6 months (T3) to detect changes after treatment.

Questionnaires were delivered via links containing both instruments, accessible on patients’ mobile devices, for WOMET and KOOS‐4 (Supporting Information). Clear instructions were provided, and responses were recorded electronically to ensure traceability and compliance.

### Statistical Analysis

All statistical analyses were performed using Jamovi software (Version 2.6, Sydney, Australia). Categorical variables are presented as frequencies and percentages and continuous variables as measures of central tendency (mean or median) and dispersion (standard deviation or interquartile range). The normality of continuous variables was assessed using the Shapiro‐Wilk test. Concomitantly, the following were analyzed.

### Reliability

Test‐retest reliability was evaluated using the intraclass correlation coefficient (ICC) with a 2‐way mixed‐effects model for absolute agreement between baseline (T0) and retest (T1) measurements. An ICC >0.90 was considered excellent.^12^, ^20^ This was complemented by Bland‐Altman plots to assess systematic bias and limits of agreement.^21^

### Internal Consistency

Internal consistency was assessed using Cronbach's *α* and McDonald's *ω* for the Spanish WOMET at baseline (T0) for both total score and individual domains. Values >0.80 were considered good and >0.90 excellent.^15^

### Construct Validity (Convergent Validity)

Convergent validity was examined by correlating WOMET and KOOS‐4 scores at baseline (T0)^15^ using the Spearman's rank correlation coefficient (because of the ordinal nature of scores) with 95% confidence intervals. Correlations >0.80 were interpreted as strong.^4^

### Precision

Precision was assessed by measuring the error inherent in the instrument. It was calculated using the standard error of measurement [SEM = standard deviation × √(1 − ICC)] and the minimal detectable change (MDC = 1.96 × √2 × SEM) at the 95% confidence level. These parameters allowed differentiation between changes observed at baseline measurement (Pe0) attributable to random measurement error (SEM) or true changes exceeding measurement error (minimal detectable change at 95% confidence level [MDC95%]).

### Floor and Ceiling Effects

The presence of floor and ceiling effects was evaluated for the total WOMET score at baseline (T0) and 6 months (T3) postoperatively. The floor effect was calculated as the proportion of patients obtaining the minimum score (0 points) and the ceiling effect as the proportion obtaining the maximum score (100 points). A floor or ceiling effect was considered substantial if more than 15% of participants achieved the lowest or highest possible score, respectively, following the recommended guidelines for patient‐reported outcome measures.^15^

### Responsiveness

Responsiveness was assessed using the standardized response mean (SRM), calculated as the mean difference between 6‐month postoperative (T3) and baseline (T0) scores divided by the standard deviation of the difference.^5^ SRM was also calculated between 3‐month postoperative (T2) and baseline (T0) scores;^15^ 95% confidence intervals were reported.

## RESULTS

A total of 110 patients were eligible for the study, recruited from 2 reference knee surgery centers in Barcelona between July and December 2025. After applying the inclusion and exclusion criteria, 39 patients were excluded (24 declined to participate, 12 were outside the predefined age range of 18 to 60 years, and 3 required revision surgery related to prior procedures). Ultimately, 71 patients were included (Figure 1), there were no losses at any time point, and all completed a 6‐month follow‐up evaluation. The mean follow‐up time was 6.1 months (range: 5.7‐6.8 months). All patients completed the questionnaires without difficulty. The Spanish WOMET showed high acceptability, with a 100% completion rate, no missing items, and a mean completion time of less than 10 minutes.

**FIGURE 1:**
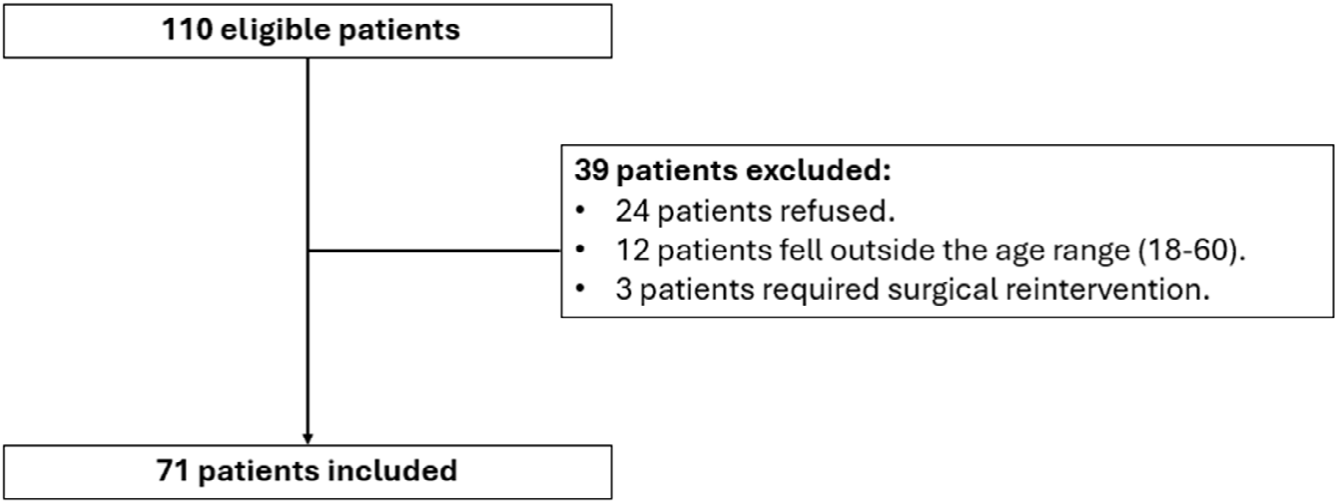
Flowchart of inclusion.

The cultural adaptation and translation of the WOMET into Spanish were accomplished without significant semantic, idiomatic, or cultural discrepancies. The expert committee and pilot testing in 30 participants confirmed the clarity, comprehensibility, and cultural relevance of all items.

Of the 71 patients included in the analysis, 67.7% were men. The median age was 47 years (interquartile range: 36.0‐53.5 years) because the distribution was non‐normal. The body mass index had a mean of 26.2 ± 3.3. Partial arthroscopic meniscectomy was performed in 85.9% of cases. Mean baseline scores (T0) for the Spanish versions of the WOMET and KOOS‐4 were 33.3 ± 13.9 and 37.5 ± 12.2, respectively (Table 1). No significant differences were observed between male and female patients in baseline WOMET scores (men: 32.8 ± 14.2, women: 34.4 ± 13.4; *P* = .68). Similarly, responsiveness (SRM) was comparable between groups (men: 2.58, women: 2.49). Detailed sex‐disaggregated data are presented in Table 2.

**TABLE 1:** Demographic and Baseline Characteristics of the Study Population (n = 71)

**Characteristics**	**N (%)**
Sex	
Male	48 (67.7)
Female	23 (32.4)
Age, yr†	47 (36.0‐53.5)
BMI∗	26.2 (±3.3)
Procedure	
Meniscectomy	61 (85.9)
Repair	10 (14.1)
WOMET (T0)	33.3 (±13.9)
KOOS‐4 (T0)	37.5 (±12.2)

BMI, body mass index; IQR, interquartile range; KOOS, Knee Injury and Osteoarthritis Outcome Score; T0, baseline measurement; WOMET, Western Ontario Meniscal Evaluation Tool; yr, years.

∗ Normally distributed data are expressed as mean ± standard deviation.

† Non‐normally distributed data are expressed as median and IQR.

**TABLE 2:** Responsiveness of the Spanish WOMET Score

**Measurement (n = 71)**	**WOMET Score (Mean ± SD)**	**Measurement vs T0 (95% CI)**	***P*** **Value**	**SRM**
T0 (basal)	33.3 (±13.9)	N/A	N/A	N/A
Male	32.8 (±14.2)	N/A	.681	2.58
Female	34.4 (±13.4)	N/A		2.49
T1 (retest)	33.0 (±13.7)	−0.35 (−1.21 to 0.50)	.416	−0.09
T2 (3 mo)	74.1 (±19.4)	40.81 (37.25‐44.37)	<.001	2.71
T3 (6 mo)	79.4 (±20.4)	46.03 (41.79‐50.29)	<.001	2.55
Meniscectomy (n = 61)	78.9 (±21.6)	44.1 (39.5‐48.7)	<.001	2.42
Repair (n = 10)	82.1 (±11.1)	56.8 (45.4‐68.2)	<.001	3.57

CI, confidence interval; mo, months; N/A, not applicable; T0, baseline measurement; T1, retest measurement 2 weeks after baseline; T2, postoperative measurement at 3 months; T3, postoperative measurement at 6 months; SD, standard deviation; SRM, standardized response mean; WOMET, Western Ontario Meniscal Evaluation Tool.

### Reliability

Test‐retest reliability analysis of the Spanish WOMET yielded an ICC of 0.96 (95% confidence interval 0.95‐0.97), indicating excellent temporal stability (Table 3). The Bland‐Altman plot for test‐retest reliability of the total WOMET score showed no systematic bias. The 95% limits of agreement were −6.7 to +7.5 points. Mild heteroscedasticity was observed, along with a nonsignificant proportional bias (slope = −0.08; *P* = .12), with no clinical relevance. Most points fell within the limits of agreement, confirming excellent agreement between the 2 preoperative assessments separated by 2 weeks (Figure 2). Symptoms remained stable during this period while awaiting surgery.

**TABLE 3:** Test‐Retest Reliability of the WOMET Score at T0 and T1 and Precision (SEM and MDC95%)

**WOMET**	**Mean ± SD**		**Reliability**			**ICC WOMET Original** ^3^	**SEM**	**MDC95%**
	**T0**	**T1**	**ICC**	**CI 95%**	***P***		**T0**	**T0**
Physical symptoms	44.1 (±17.7)	43.8 (±18.2)	0.97	0.95‐0.98	<.001	0.73	3.06	8.46
Sports/recreation/work/lifestyle	20.1 (±13.6)	20.0 (±14.0)	0.95	0.92‐0.97	<.001	0.87	3.04	8.40
Emotions	18.6 (±11.4)	17.7 (±12.0)	0.88	0.81‐0.92	<.001	0.84	3.94	10.89
Total WOMET score	33.3 (±13.9)	33.0 (±13.7)	0.96	0.94‐0.97	<.001	0.85	2.78	7.68
Total KOOS‐4 score	37.5 (±12.2)	37.1 (±11.8)	0.95	0.93‐0.97	<.001		2.72	7.52

CI, confidence interval; ICC, intraclass correlation coefficient; KOOS, Knee Injury and Osteoarthritis Outcome Score; MDC95%, minimal detectable change at 95% confidence level; SD, standard deviation; SEM, standard error of measurement; T0, baseline measurement; T1, retest measurement; WOMET, Western Ontario Meniscal Evaluation Tool.

**FIGURE 2:**
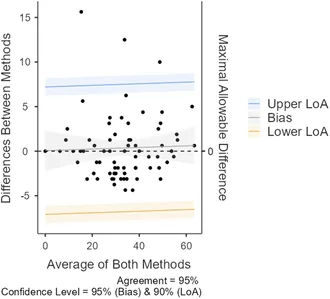
Bland‐Altman plot of test‐retest reliability of the Spanish WOMET total score (n = 71). (LoA, limits of agreement; WOMET, Western Ontario Meniscal Evaluation Tool.)

### Internal Consistency

Internal consistency analysis of the Spanish WOMET revealed a Cronbach's *α* of 0.90 and a McDonald's *ω* of 0.92 at baseline (T0), showing high internal validity. For the WOMET subscales, Cronbach's *α* ranged from 0.87 to 0.89 and McDonald's *ω* from 0.89 to 0.90. Individual item deletion did not significantly affect the internal consistency at baseline (T0) (Table 4).

**TABLE 4:** Internal Consistency of the WOMET Score at Baseline T0

**Score/Domain**	**Cronbach's** ***α***	**McDonald's** ***ω***	**Cronbach's** ***α*** **if Item Deleted**
Physical symptoms	0.89	0.90	0.87‐0.90
Sports/recreation/work/lifestyle	0.87	0.89	0.80‐0.91
Emotions	0.88	0.89	0.81‐0.87
Total WOMET score	0.90	0.92	0.89‐0.91
Total KOOS‐4 score	0.88	0.92	0.87‐0.91

KOOS, Knee Injury and Osteoarthritis Outcome Score; T0, baseline measurement; WOMET, Western Ontario Meniscal Evaluation Tool.

### Construct Validity (Convergent Validity)

Spearman's correlation between total scores of the Spanish WOMET and Spanish KOOS‐4 at baseline (T0) was 0.90 (*P* < .05), indicating a strong correlation. Spearman's rho coefficients for conceptually similar domains showed statistically significant convergent correlations (*P* < .05) (Figure 3).

**FIGURE 3:**
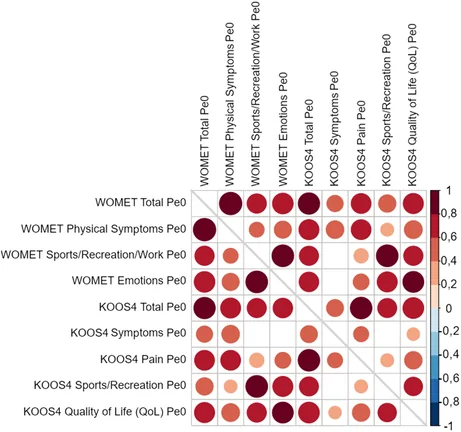
Heatmap of Spearman's rank correlation coefficients between the Spanish version of the WOMET and KOOS‐4 domains at baseline evaluation (n = 71). Larger and darker red circles indicate stronger positive correlations. All correlations (circles) were statistically significant (*P* < .05). (KOOS, Knee Injury and Osteoarthritis Outcome Score; WOMET, Western Ontario Meniscal Evaluation Tool.)

### Precision

The Spanish WOMET showed an SEM of 2.78 at T0. The emotions subdomain had the highest SEM compared with the other subdomains (3.94). The MDC95% was 7.68 at T0 (Table 3).

### Floor and Ceiling Effects

For the total Spanish WOMET score, at baseline, no floor (0%) or ceiling (0%) effects were observed. At 6‐month follow‐up, the floor effect remained 0%, whereas a minimal ceiling effect was detected (4.23%, n = 3).

### Responsiveness

SRM was calculated by comparing baseline (T0) scores of the Spanish WOMET with subsequent measurements (T1, T2, and T3). Comparison between T1 and T0 yielded an SRM of −0.09, with no statistically significant difference (*P* = .416), confirming temporal stability. At 3 months (T2 vs T0) and 6 months (T3 vs T0), the SRM values were 2.71 and 2.55, respectively, with significant differences (*P* < .001). When stratified by procedure type at 6 months (T3), the SRM was 2.42 in the meniscectomy group and 3.57 in the meniscal repair group (Table 2).

## DISCUSSION

This study validated the Spanish version of the WOMET questionnaire in patients undergoing arthroscopic surgery for meniscal tears at a minimum 6‐month follow‐up, showing excellent psychometric properties: high reliability (ICC: 0.96), elevated internal consistency (Cronbach's *α*: 0.90, McDonald's *ω*: 0.92), strong convergent validity with the KOOS‐4 (Spearman's rho =0.90; *P* < .05), good precision (SEM: 2.78, MDC95%: 7.68), and substantial responsiveness to change (SRM: 2.71 and 2.56 at 3 and 6 months, respectively). These findings are comparable or superior to those reported in cross‐cultural adaptations to other languages, including Italian (ICC: 0.87),^10^ German (ICC: 0.90),^4^ Turkish (ICC: 0.88),^6^ Danish (ICC: 0.88),^7^ Dutch (ICC: 0.78),^5^ and Chinese (ICC: 0.93).^8^ The test‐retest reliability was excellent and superior to most published versions (ICC range: 0.78‐0.93), confirming the temporal stability of the WOMET in our cultural and health care context.

The internal consistency of the Spanish WOMET yielded a Cronbach's *α* of 0.90, which is at the upper end of reported values. The inclusion of McDonald's *ω* (0.92) further strengthens the robustness of the measurement because it is based on a more realistic factor model than Cronbach's *α*. Most previous studies assessed only Cronbach's *α*, as seen in the Dutch (alpha: 0.87),^5^ German (alpha: 0.92),^4^ and Finnish (alpha: 0.91)^9^ adaptations, which are comparable to our results. This high Cronbach's *α* may be explained by the cultural and linguistic adaptation process, which ensured that all items were clear, relevant, and well‐understood by Spanish‐speaking patients.

The strong correlation between the WOMET and KOOS‐4 (rho = 0.90) supports convergent validity and suggests that the Spanish WOMET not only effectively captures meniscal pathology but also reliably reflects overall knee status, consistent with other validations that compared it with generic knee scores (Lysholm and International Knee Documentation Committee).^10^ The WOMET is a disease‐specific tool designed exclusively for meniscal pathology. This specificity likely explains its stronger ability to capture the impact of meniscal tears on physical symptoms, sports/recreation/work, and emotional aspects, resulting in excellent convergent validity with the KOOS‐4 while offering greater sensitivity for this specific condition.

Precision of the Spanish WOMET was high, with an SEM of 2.78 and an MDC95% of 7.68 values lower than those reported for the Dutch (SEM: 7.4, MDC: 14.7)^5^ and Danish (SEM: 8, MDC: 22)^7^ versions, indicating greater sensitivity in detecting clinically relevant changes with a smaller margin of measurement error in meniscal pathology.

Responsiveness to change of the Spanish WOMET was substantial, with SRM values of 2.56 to 2.71, exceeding those of the Italian (SRM: 1.94‐2.44),^10^ Danish (SRM: 1.12),^7^ Finnish (SRM: 1.17),^9^ and Chinese (SRM: 1.11) versions.^8^ This high SRM may be related to several factors, including the high quality of the cross‐cultural adaptation, the electronic administration format (which may reduce missing data and improve completion), and the characteristics of our surgical cohort, in which most patients experienced substantial clinical improvement after arthroscopic treatment. This finding is particularly relevant in the context of meniscal surgery, where functional and emotional changes after intervention can be considerable, underscoring the utility of this questionnaire for postoperative follow‐up and evaluation of therapeutic effectiveness. Although the Spanish WOMET showed high responsiveness in both surgical groups, the repair subgroup was small (n = 10; 14.1% of the cohort). Therefore, although excellent responsiveness was observed in patients undergoing meniscal repair (SRM = 3.57), this finding should be interpreted cautiously. The overall results primarily reflect outcomes after partial meniscectomy, which represented most cases (85.9%). The minimal clinically important difference was not calculated because no external anchor (e.g., global rating of change) was included in the study, and the large SRM values (2.71 at 3 months and 2.55 at 6 months) far exceed the commonly accepted threshold for large effect size (>0.8), indicating a substantial and clinically meaningful improvement after surgery.

Meniscal pathology remains one of the most common conditions in contemporary knee surgery practice. The availability of a specific, comprehensible, brief (16 items), well‐accepted, and psychometrically robust (reliable, valid, and precise) instrument represents a clear clinical advantage of the WOMET over longer alternatives such as the KOOS‐4 (25 items) or generic scores like the International Knee Documentation Committee or Lysholm. This work fills an important knowledge gap in our setting. No difficulties were encountered in questionnaire completion, and patient acceptability was excellent.

### Limitations

Several limitations should be acknowledged. The study was conducted at only 2 reference centers, which may limit generalizability, particularly to other Spanish‐speaking populations with cultural diversity, such as Latin American groups, where semantic differences exist despite shared language. In accordance with Sex and Gender Equity in Research guidelines, a sex‐disaggregated analysis was performed. However, the small number of female participants (n = 23) limits the statistical power for subgroup comparisons. Additionally, although international guidelines and the Consensus‐based Standards for the selection of health Measurement Instruments checklist were followed, measurement invariance was not formally tested, and the sample size (n = 71) is below the recommended threshold of 100 for some psychometric analyses, particularly structural validity.^22^ Although the study followed a consecutive recruitment approach, potential selection bias may still exist because 24 of 110 eligible patients (21.8%) declined participation. Patients who consented might have been more motivated or had milder symptoms, potentially affecting the generalizability of the findings. Follow‐up was limited to 6 months; although the study included both partial meniscectomy and meniscal repair patients, the repair group was small (n = 10, 14.1%) and the follow‐up was limited to 6 months. This duration is adequate for meniscectomy but may be insufficient for meniscal repair; therefore, the excellent responsiveness observed in the repair subgroup should be interpreted with caution. An electronic administration method was used (consistent with current trends), but it is unknown whether paper‐based administration might yield different results. The performance of the questionnaire in conservatively managed meniscal lesions was not evaluated, which could provide a more comprehensive perspective.

## CONCLUSIONS

The Spanish version of the WOMET questionnaire is a reliable, valid, precise, and highly responsive instrument for assessing clinical outcomes in patients with meniscal pathology treated surgically in Spanish‐speaking patients. Its brevity (16 items), specificity, and excellent patient acceptability make it an ideal tool for routine clinical practice and research.

## SUPPORTING INFORMATION

Additional supporting information can be found online in the Supporting Information section.

## DISCLOSURES

The authors (M.A.P‐F., M.I., Z.L.W., N.P., R.T‐C., S.P., J.C.M.) declare that they have no known competing financial interests or personal relationships that could have appeared to influence the work reported in this paper.

## Supporting information

Supplementary Material

Supplementary Material
