# Supplementary material for: The Spanish Version of the Western Ontario Meniscal Evaluation Tool Is a Reliable, Valid, Precise, and Highly Responsive Instrument for Patients With Meniscal Pathology at a Minimum 6‐Month Follow‐Up
Source: Arthrosc Sports Med Rehabil. 2026 Oct 23:e70131. Online ahead of print. doi: 10.1002/ars2.70131 (PMC13692319; doi:10.1002/ars2.70131)
Supplement: Supplementary file 1 [file ARS2-9999-e70131-s001.pdf]

Escala de valoración KOOS 4

Buen dia:
Esta encuesta r**ecoge su opinión sobre su rodilla intervenida o lesionada**. La información que nos proporcione, servirá para saber como se encuentra y la capacidad para realizar diferentes actividades.

Responda a cada pregunta marcando la casilla apropiada y solo una casilla por pregunta. Señale siempre la respuesta que **mejor refleja su situación**.

Por favor, marque el número que mejor describa cómo se ha sentido.

* Indica que la pregunta es obligatoria

1. Correo *

2. Apellidos y Nombres *

3. Fecha en que llena este formulario *

   Ejemplo: 7 de enero del 2019

4. Genero *

   Marca solo un óvalo.

   - Masculino
   - Femenino
   - Otro

5. Edad *

6. ¿Es la primera vez que llena este cuestionario? *

Marca solo un óvalo.

- Primera vez (Pe0)
- Segunda vez (Pe1)
- Tercera vez (Po2)
- Cuarta vez (Po3)

A. Síntomas

Responda a estas preguntas considerando los síntomas que ha notado en la rodilla durante la última semana:

7. S1. ¿Se le hincha la rodilla? *

Marca solo un óvalo.

- Nunca
- Rara vez
- A veces
- Frecuentemente
- Siempre

8. S2. ¿Siente crujidos, chasquidos u otro tipo de ruidos cuando mueve la rodilla? *

Marca solo un óvalo.

- Nunca
- Rara vez
- A veces
- Frecuentemente
- Siempre

9. S3. Al moverse, ¿Siente que la rodilla falla o se bloquea? *

Marca solo un óvalo.

- Nunca
- Rara vez
- A veces
- Frecuentemente
- Siempre

10. S4. ¿Puede estirar completamente la rodilla? *

Marca solo un óvalo.

- Siempre
- Frecuentemente
- A veces
- Rara vez
- Nunca

11. S5. ¿Puedo doblar completamente la rodilla? *

Marca solo un óvalo.

- Siempre
- Frecuentemente
- A veces
- Rara vez
- Nunca

A. Rigidez Articular

La rigidez o entumecimiento es una sensación de limitación o lentitud en el movimiento de la rodilla. Las siguientes preguntas indagan el grado de rigidez que ha experimentado, en la rodilla, durante la última semana.

12. S6. ¿Cuál es el grado de rigidez de su rodilla al levantarse por la mañana? *

Marca solo un óvalo.

- No tengo
- Leve
- Moderado
- Intenso
- Muy intenso

13. S7. ¿Cuál es el grado de rigidez de la rodilla después de estar sentado, recostado o descansando? *

Marca solo un óvalo.

- No tengo
- Leve
- Moderado
- Intenso
- Muy intenso

B. Dolor

Las siguientes preguntas indagan el grado de dolor que ha experimentado, en la rodilla:

14. D1. ¿Con qué frecuencia ha tenido dolor en su rodilla? *

Marca solo un óvalo.

- Nunca
- Mensual
- Semanal
- Diario
- Continuo

15. D2. ¿Cuánto dolor ha tenido en la rodilla en la última semana al girar o pivotar sobre su rodilla? *

Marca solo un óvalo.

- No tengo
- Leve
- Moderado
- Intenso
- Muy Intenso

16. D3. ¿Cuánto dolor ha tenido en la rodilla en la última semana al estirar completamente la rodilla? *

Marca solo un óvalo.

- No tengo
- Leve
- Moderado
- Intenso
- Muy Intenso

17. D4. ¿Cuánto dolor ha tenido en la rodilla en la última semana al doblar completamente la rodilla? *

Marca solo un óvalo.

- No tengo
- Leve
- Moderado
- Intenso
- Muy Intenso

18. D5. ¿Cuánto dolor ha tenido en la rodilla en la última semana al caminar, sobre una superficie plana? *

Marca solo un óvalo.

- No tengo
- Leve
- Moderado
- Intenso
- Muy Intenso

19. D6. ¿Cuánto dolor ha tenido en la rodilla en la última semana al subir o bajar escaleras? *

Marca solo un óvalo.

- No tengo
- Leve
- Moderado
- Intenso
- Muy Intenso

20. D7. ¿Cuánto dolor ha tenido en la rodilla en la última semana por la noche, en la cama? *

Marca solo un óvalo.

- No tengo
- Leve
- Moderado
- Intenso
- Muy Intenso

21. D8. ¿Cuánto dolor ha tenido en la rodilla en la última semana al estar sentado o recostado? *

Marca solo un óvalo.

- No tengo
- Leve
- Moderado
- Intenso
- Muy Intenso

22. D9. ¿Cuánto dolor ha tenido en la rodilla en la última semana al estar de pie? *

Marca solo un óvalo.

- No tengo
- Leve
- Moderado
- Intenso
- Muy Intenso

C. Función, actividades deportivas y recreacionales

Las siguientes preguntas indagan sobre su función al realizar actividades que requieran un mayor nivel de esfuerzo. Las preguntas deben responderse pensando en el grado de dificultad experimentado con su rodilla, en la última semana:

23. SP1. ¿Tiene dificultad al ponerse en cuclillas? *

Marca solo un óvalo.

- No tengo
- Leve
- Moderado
- Intenso
- Muy Intenso

24. SP2. ¿Tiene dificultad al correr? *

Marca solo un óvalo.

- No tengo
- Leve
- Moderado
- Intenso
- Muy Intenso

25. SP3. ¿Tiene dificultad al saltar? *

Marca solo un óvalo.

- No tengo
- Leve
- Moderado
- Intenso
- Muy Intenso

26. SP4. ¿Tiene dificultad al girar o pivotar sobre la rodilla afectada? *

Marca solo un óvalo.

- No tengo
- Leve
- Moderado
- Intenso
- Muy Intenso

27. SP5. ¿Tiene dificultad al arrodillarse? *

Marca solo un óvalo.

- No tengo
- Leve
- Moderado
- Intenso
- Muy Intenso

D. Calidad de vida

Responder como impacta el problema de su rodilla sobre su calidad de vida:

28. Q1. ¿Con qué frecuencia es consciente del problema de su rodilla? *

Marca solo un óvalo.

- Nunca
- Mensualmente
- Semanalmente
- A diario
- Siempre

29. Q2. ¿Ha modificado su estilo de vida para evitar actividades que puedan lesionar su rodilla? *

Marca solo un óvalo.

- No
- Levemente
- Moderadamente
- Drasticamente
- Totalmente

30. Q3. ¿En qué medida está preocupado por la falta de seguridad en su rodilla? *

Marca solo un óvalo.

- Nunca
- Levemente
- Moderadamente
- Mucho
- Excesivamente

31. Q4. En general, ¿cuántas dificultades le crea su rodilla? *

Marca solo un óvalo.

- Ninguna
- Algunas
- Pocas
- Muchas
- Todas

Este contenido no ha sido creado ni aprobado por Google.

Google Formularios
