# Supplementary material for: The Spanish Version of the Western Ontario Meniscal Evaluation Tool Is a Reliable, Valid, Precise, and Highly Responsive Instrument for Patients With Meniscal Pathology at a Minimum 6‐Month Follow‐Up
Source: Arthrosc Sports Med Rehabil. 2026 Oct 23:e70131. Online ahead of print. doi: 10.1002/ars2.70131 (PMC13692319; doi:10.1002/ars2.70131)
Supplement: Supplementary file 2 [file ARS2-9999-e70131-s002.pdf]

Escala de valoracion WOMET

Buen dia:

A continuación encontrará una serie de preguntas sobre su estado de salud en la ultima semana. Para responder, deberá marcar un número del 0 al 10 según cómo se sienta.

- El número **0** significa que **no tiene dolor, molestias o problemas** relacionados con la pregunta.
- El número **10** significa que **el dolor, las molestias o los problemas son lo más intensos posibles**.

Por favor, marque el número que mejor describa cómo se ha sentido **recientemente**.

* Indica que la pregunta es obligatoria

1. Correo *

2. Apellidos y nombres *

3. Fecha en que llena este formulario *

Ejemplo: 7 de enero del 2019

4. Genero *

Marca solo un óvalo.

- Masculino
- Femenino
- Otro

5. Edad *

6. ¿Es la primera vez que llena este cuestionario? *

Marca solo un óvalo.

- Primera vez (Pe0)
- Segunda vez (Pe1)
- Tercera vez (Po2)
- Cuarta vez (Po3)

Escala de valoracion WOMET

7. Apellidos y nombres *

8. Fecha en que llena este formulario *

Ejemplo: 7 de enero del 2019

9. Genero *

Marca solo un óvalo.

- Masculino
- Femenino
- Otro

10. Edad *

11. ¿Es la primera vez que llena este cuestionario? *

Marca solo un óvalo.

- Primera vez (Pe0)
- Segunda vez (Pe1)
- Tercera vez (Po2)
- Cuarta vez (Po3)

A. Sintomas Fisicos

Las siguientes preguntas se refieren a los síntomas físicos que ha tenido debido a su problema de rodilla. En todos los casos, por favor indique cuánto ha sentido ese síntoma durante la última semana.

12. 1A. ¿Qué tanto le molesta la sensación de que su rodilla sea inestable o que no le de seguridad? *

Marca solo un óvalo.

	0	1	2	3	4	5	6	7	8	9	10	
Nad	◯	◯	◯	◯	◯	◯	◯	◯	◯	◯	◯	Extremadamente incomodo

13. 2A. ¿Cuánto le molesta el dolor o la sensibilidad en su rodilla después de hacer alguna actividad? *

Marca solo un óvalo.

	0	1	2	3	4	5	6	7	8	9	10	
Nad	◯	◯	◯	◯	◯	◯	◯	◯	◯	◯	◯	Extremadamente incomodo

14. 3A. ¿Cuánto le molesta sentir que ha perdido rango de movimiento en su rodilla? *

Marca solo un óvalo.

	0	1	2	3	4	5	6	7	8	9	10	
Nad	◯	◯	◯	◯	◯	◯	◯	◯	◯	◯	◯	Extremadamente incomodo

15. 4A. ¿Cuánto le molesta sentir adormecida la rodilla o los alrededores de la misma? *

Marca solo un óvalo.

	0	1	2	3	4	5	6	7	8	9	10	
Nad	◯	◯	◯	◯	◯	◯	◯	◯	◯	◯	◯	Extremadamente incomodo

16. 5A. ¿Cuánto le molesta sentir su rodilla rigida al levantarse en la mañana o después de estar sentado mucho tiempo? *

Marca solo un óvalo.

	0	1	2	3	4	5	6	7	8	9	10	
Nad	◯	◯	◯	◯	◯	◯	◯	◯	◯	◯	◯	Extremadamente incomodo

17. 6A. ¿Cuánto le molesta sentir que su rodilla está débil o sin fuerza? *

Marca solo un óvalo.

	0	1	2	3	4	5	6	7	8	9	10	
Nad	◯	◯	◯	◯	◯	◯	◯	◯	◯	◯	◯	Extremadamente incomodo

18. 7A. ¿Cuánto le molesta la hinchazón en su rodilla? *

Marca solo un óvalo.

	0	1	2	3	4	5	6	7	8	9	10	
Nad	◯	◯	◯	◯	◯	◯	◯	◯	◯	◯	◯	Extremadamente incomodo

19. 8A. ¿Cuánto le molestan los dolores agudos en su rodilla después de estar mucho tiempo apoyando todo el peso en ella? *

Marca solo un óvalo.

	0	1	2	3	4	5	6	7	8	9	10	
Nad	◯	◯	◯	◯	◯	◯	◯	◯	◯	◯	◯	Extremadamente incomodo

20. 9A. ¿Cuánto le molestan los ruidos en su rodilla como crujidos, crepitos (raspados) o chasquidos ? *

Marca solo un óvalo.

	0	1	2	3	4	5	6	7	8	9	10	
Nad	◯	◯	◯	◯	◯	◯	◯	◯	◯	◯	◯	Extremadamente incomodo

B. Deportes/recreacion/trabajo/vida diaria

La siguiente sección se refiere a cómo su problema de rodilla ha afectado su trabajo, actividades deportivas o recreativas durante la última semana.

21. 10B. ¿Cuánto miedo tiene de volver a lesionarse la rodilla si regresa a su trabajo o deporte? *

Marca solo un óvalo.

	0	1	2	3	4	5	6	7	8	9	10	
Nad	◯	◯	◯	◯	◯	◯	◯	◯	◯	◯	◯	Extremo temor

22. 11B. ¿Cuánto ha afectado la lesion en su rodilla, al tiempo que dedica a las actividades que hacía antes de su lesion? *

Marca solo un óvalo.

	0	1	2	3	4	5	6	7	8	9	10	
Nad	◯	◯	◯	◯	◯	◯	◯	◯	◯	◯	◯	Extremadamente afectado

23. 12B. ¿Cuánto ha afectado su rodilla la capacidad para hacer bien las tareas que necesita en su trabajo o deporte? *

Marca solo un óvalo.

	0	1	2	3	4	5	6	7	8	9	10	
Nad	◯	◯	◯	◯	◯	◯	◯	◯	◯	◯	◯	Extremadamente afectado

24. 13B. ¿Qué tanto problema tiene para agacharse o ponerse en cuclillas? *

Marca solo un óvalo.

	0	1	2	3	4	5	6	7	8	9	10	
Nad	◯	◯	◯	◯	◯	◯	◯	◯	◯	◯	◯	Extremadamente problematico

C. Emociones

Las siguientes preguntas se refieren a cómo se ha sentido durante la última semana en relación con su problema de rodilla.

25. 14C. ¿Qué tanto está pendiente o se da cuenta de su rodilla? *

Marca solo un óvalo.

	0	1	2	3	4	5	6	7	8	9	10	
Nad	◯	◯	◯	◯	◯	◯	◯	◯	◯	◯	◯	Extremadamente conciente

26. 15C. ¿Qué tanto le preocupa lo que pueda pasar con su rodilla más adelante? *

Marca solo un óvalo.

	0	1	2	3	4	5	6	7	8	9	10	
Nad	◯	◯	◯	◯	◯	◯	◯	◯	◯	◯	◯	Extremadamente preocupado

27. 16C. ¿Qué tanto se siente frustrado o desanimado por culpa de su rodilla? *

Marca solo un óvalo.

	0	1	2	3	4	5	6	7	8	9	10	
Nad	◯	◯	◯	◯	◯	◯	◯	◯	◯	◯	◯	Extremadamente

A. Sintomas Fisicos

Las siguientes preguntas se refieren a los síntomas físicos que ha tenido debido a su problema de rodilla. En todos los casos, por favor indique cuánto ha sentido ese síntoma durante la última semana.

28. 1A. ¿Qué tanto le molesta la sensación de que su rodilla sea inestable o que no le de seguridad? *

Marca solo un óvalo.

	0	1	2	3	4	5	6	7	8	9	10	
Nad	◯	◯	◯	◯	◯	◯	◯	◯	◯	◯	◯	Extremadamente incomodo

29. 2A. ¿Cuánto le molesta el dolor o la sensibilidad en su rodilla después de hacer alguna actividad? *

Marca solo un óvalo.

	0	1	2	3	4	5	6	7	8	9	10	
Nad	◯	◯	◯	◯	◯	◯	◯	◯	◯	◯	◯	Extremadamente incomodo

30. 3A. ¿Cuánto le molesta sentir que ha perdido rango de movimiento en su rodilla? *

Marca solo un óvalo.

	0	1	2	3	4	5	6	7	8	9	10	
Nad	◯	◯	◯	◯	◯	◯	◯	◯	◯	◯	◯	Extremadamente incomodo

31. 4A. ¿Cuánto le molesta sentir adormecida la rodilla o los alrededores de la misma? *

Marca solo un óvalo.

	0	1	2	3	4	5	6	7	8	9	10	
Nad	◯	◯	◯	◯	◯	◯	◯	◯	◯	◯	◯	Extremadamente incomodo

32. 5A. ¿Cuánto le molesta sentir su rodilla rigida al levantarse en la mañana o después de estar sentado mucho tiempo? *

Marca solo un óvalo.

	0	1	2	3	4	5	6	7	8	9	10	
Nad	◯	◯	◯	◯	◯	◯	◯	◯	◯	◯	◯	Extremadamente incomodo

33. 6A. ¿Cuánto le molesta sentir que su rodilla está débil o sin fuerza? *

Marca solo un óvalo.

	0	1	2	3	4	5	6	7	8	9	10	
Nad	◯	◯	◯	◯	◯	◯	◯	◯	◯	◯	◯	Extremadamente incomodo

34. 7A. ¿Cuánto le molesta la hinchazón en su rodilla? *

Marca solo un óvalo.

	0	1	2	3	4	5	6	7	8	9	10	
Nad	◯	◯	◯	◯	◯	◯	◯	◯	◯	◯	◯	Extremadamente incomodo

35. 8A. ¿Cuánto le molestan los dolores agudos en su rodilla después de estar mucho tiempo apoyando todo el peso en ella? *

Marca solo un óvalo.

	0	1	2	3	4	5	6	7	8	9	10	
Nad	◯	◯	◯	◯	◯	◯	◯	◯	◯	◯	◯	Extremadamente incomodo

36. 9A. ¿Cuánto le molestan los ruidos en su rodilla como crujidos, crepitos (raspados) o chasquidos ? *

Marca solo un óvalo.

	0	1	2	3	4	5	6	7	8	9	10	
Nad	◯	◯	◯	◯	◯	◯	◯	◯	◯	◯	◯	Extremadamente incomodo

B. Deportes/recreacion/trabajo/vida diaria

La siguiente sección se refiere a cómo su problema de rodilla ha afectado su trabajo, actividades deportivas o recreativas durante la última semana.

37. 10B. ¿Cuánto miedo tiene de volver a lesionarse la rodilla si regresa a su trabajo o deporte? *

Marca solo un óvalo.

	0	1	2	3	4	5	6	7	8	9	10	
Nad	◯	◯	◯	◯	◯	◯	◯	◯	◯	◯	◯	Extremo temor

38. 11B. ¿Cuánto ha afectado la lesion en su rodilla, al tiempo que dedica a las actividades que hacía antes de su lesion? *

Marca solo un óvalo.

	0	1	2	3	4	5	6	7	8	9	10	
Nad	◯	◯	◯	◯	◯	◯	◯	◯	◯	◯	◯	Extremadamente afectado

39. 12B. ¿Cuánto ha afectado su rodilla la capacidad para hacer bien las tareas que necesita en su trabajo o deporte? *

Marca solo un óvalo.

	0	1	2	3	4	5	6	7	8	9	10	
Nad	◯	◯	◯	◯	◯	◯	◯	◯	◯	◯	◯	Extremadamente afectado

40. 13B. ¿Qué tanto problema tiene para agacharse o ponerse en cuclillas? *

Marca solo un óvalo.

	0	1	2	3	4	5	6	7	8	9	10	
Nad	◯	◯	◯	◯	◯	◯	◯	◯	◯	◯	◯	Extremadamente problematico

C. Emociones

Las siguientes preguntas se refieren a cómo se ha sentido durante la última semana en relación con su problema de rodilla.

41. 14C. ¿Qué tanto está pendiente o se da cuenta de su rodilla? *

Marca solo un óvalo.

	0	1	2	3	4	5	6	7	8	9	10	
Nad	◯	◯	◯	◯	◯	◯	◯	◯	◯	◯	◯	Extremadamente conciente

42. 15C. ¿Qué tanto le preocupa lo que pueda pasar con su rodilla más adelante? *

Marca solo un óvalo.

	0	1	2	3	4	5	6	7	8	9	10	
Nad	◯	◯	◯	◯	◯	◯	◯	◯	◯	◯	◯	Extremadamente preocupado

43. 16C. ¿Qué tanto se siente frustrado o desanimado por culpa de su rodilla? *

Marca solo un óvalo.

	0	1	2	3	4	5	6	7	8	9	10	
Nad	◯	◯	◯	◯	◯	◯	◯	◯	◯	◯	◯	Extremadamente

Este contenido no ha sido creado ni aprobado por Google.

Google Formularios
